# Is it possible to identify populations experiencing material disadvantage in primary care? A feasibility study using the Clinical Practice Research Database

**DOI:** 10.1136/jech-2024-222396

**Published:** 2024-09-03

**Authors:** Laurie E Davies, David R Sinclair, Andrew Kingston, Gemma Frances Spiers, Barbara Hanratty

**Affiliations:** 1 National Institute for Health and Care Research (NIHR) Policy Research Unit in Older People and Frailty/Healthy Ageing, Population Health Sciences Institute, Newcastle University, Newcastle upon Tyne, UK

**Keywords:** PRIMARY HEALTH CARE, POVERTY, Health inequalities, GENERAL PRACTICE, PUBLIC HEALTH

## Abstract

**Background:**

Material disadvantage is associated with poor health, but commonly available area-based metrics provide a poor proxy for it. We investigate if a measure of material disadvantage could be constructed from UK primary care electronic health records.

**Methods:**

Using data from Clinical Practice Research Datalink Aurum (May 2022) linked to the 2019 English Index of Multiple Deprivation (IMD), we sought to (1) identify codes that signified material disadvantage, (2) aggregate these codes into a binary measure of material disadvantage and (3) compare the proportion of people with this binary measure against IMD quintiles for validation purposes.

**Results:**

We identified 491 codes related to benefits, employment, housing, income, environment, neglect, support services and transport. Participants with one or more of these codes were defined as being materially disadvantaged. Among 30,897,729 research-acceptable patients aged ≥18 with complete data, only 6.1% (n=1,894,225) were classified as disadvantaged using our binary measure, whereas 42.2% (n=13,038,085) belonged to the two most deprived IMD quintiles.

**Conclusion:**

Data in a major primary care research database do not currently contain a useful measure of individual-level material disadvantage. This represents an omission of one of the most important health determinants. Consideration should be given to creating codes for use by primary care practitioners.

**WHAT IS ALREADY KNOWN ON THIS TOPIC?**

**WHAT THIS STUDY ADDS**

- There is no dedicated code for material disadvantage in UK primary care, and codes that indicate material disadvantage are sparsely recorded.

**HOW THIS STUDY MIGHT AFFECT RESEARCH, PRACTICE OR POLICY**

- Creating a consensus code to identify persons experiencing material disadvantage in primary care would be feasible and might help to improve population health.
- The potential consequences of such a code need to be sensitively considered to ensure that the benefits outweigh any costs at an individual level.

## Introduction

 The debate over the best measure of socioeconomic status remains unresolved,^1^ yet being poor or disadvantaged is one of the most important health determinants.^2 3^ Compared to the rich, the poor are more likely to experience worse health outcomes, and from earlier in their life course.^4^ Area-level indicators of disadvantage have limitations,^5^ such as the ecological fallacy, and an arbitrary threshold for poverty overlooks the important impact of relative inequality.^6^ Identifying individuals experiencing material disadvantage is crucial for addressing social determinants of health and improving health equity. Policies to improve health address a range of basic human needs rather than healthcare alone. A practical measure of an individual’s material circumstances (eg, resources such as income, housing, goods, cars and food quality) in routine datasets would therefore be useful. Electronic health records provide a digital version of a patients’ medical history, to improve patient care and outcomes. Moreover, since general practice is generally the first point of contact for health issues, the information in electronic healthcare records could describe the material circumstances of patients. Structured patient data facilitate monitoring of at-risk populations and potentially, targeting of health promotion activity based on patient characteristics. This study explores the feasibility of using routine primary care data, accessed through a research database, to identify people experiencing material disadvantage.

## Methods

The Clinical Practice Research Datalink (CPRD) routinely collates patient data from general practitioner (GP) practices across the UK. Using data from the May 2022 build of CPRD Aurum,^7^ we quantified the prevalence of material disadvantage, as a binary variable, among 30,897,729 research-acceptable patients aged 18–90, who were alive or died after 2010, and had complete 2019-IMD-linked data (available to participants registered in England only). To refine our sample, we also excluded practices that had merged with other contributing practices to avoid duplication of data.^8^ To create our binary indicator of material disadvantage, we aggregated 491 codes related to benefits, employment, housing, income, environment, neglect, support services, transport and other socioeconomic measures (online supplemental appendix 1). Participants were classified as disadvantaged if at least one of these codes was present in their electronic records. Certain terms that did not necessarily represent material disadvantage were retained out of caution. We included participants aged 18–90 in our analysis because socioeconomic inequalities in health are most pronounced in middle and early old age, and remain notable, to a lesser extent, in later old age.^3 9^ We validated our definition of material disadvantage against the IMD, which associates levels of disadvantage with specific postcodes across England. We compared the proportion of people classified as disadvantaged using our measure to those in the two most deprived IMD quintiles (4 and 5). We used quintiles 4 and 5 because the most deprived quintile in CPRD is under-represented according to Office for National Statistics population estimates.^10^ All analyses were conducted in R software, V.3.6.0.

### Ethics approval

This study was approved by CPRD’s Research Data Governance Process for Medicines and Healthcare Products Regulatory Agency Database Research (study reference ID: 23_003085). CPRD has ethics approval from the Health Research Authority to support research using anonymised patient data from consenting general practice electronic health records; therefore, additional ethical approval was not required for this observational study.^11^

## Results

In our study population, 30,897,729 participants, 42.2% (n=13,038,085) belonged to the two most deprived IMD quintiles (4 and 5). However, only 6.1% (n=1,894,225) were classified as disadvantaged across all quintiles using our binary indicator, and within the most deprived quintiles, this figure is even lower at 3.5% (table 1). These results indicate that our binary indicator may underestimate the true prevalence of material deprivation.

**Table 1:** Percentage of people experiencing our binary measure of material disadvantage compared with IMD

Material disadvantage	2019 IMD quintile 1	2019 IMD quintile 2	2019 IMD quintile 3	2019 IMD quintile 4	2019 IMD quintile 5	Sum
No	17.8	18.4	18.9	20.9	17.9	93.9
Yes	0.7	0.9	1.0	1.5	2.0	6.1
Sum	18.5	19.3	20.0	22.3	19.9	100.0

IMDIndex of Multiple Deprivation

## Discussion

### Principle findings

The current system of coding in primary care electronic health records is inadequate for assessing material disadvantage at the individual level.

### Comparison with existing literature

Effective policies to improve the public health must address the wider determinants of health, as they have a far greater influence on health than clinical care, behaviours or genetics.^12^ In the UK, all National Health Service patients must be registered with a GP.^13^ This means that electronic healthcare records offer very high levels of population coverage. Appropriately coded data could be invaluable to target health policies and monitor progress. However, there is no dedicated code for material disadvantage in UK primary care electronic health records. There are a number of reasons why codes that indicate material disadvantage are sparsely recorded in general practice. First, primary care services are likely to be taking deprivation into account in their management of patients, without recording any relevant codes.^14^ For example, for practices located in high-deprivation areas, where the majority of the population experiences material disadvantage, clinicians may be less likely to record observations of such disadvantage. Second, there is likely to be variation in the level of interest that GPs have in their patients’ social circumstances.^15^ High demands on practices in poorer areas and shorter consultations may also impact coding behaviour.^15^,^17^

### Strengths and limitations

To our knowledge, this is the first study to assess the recording of codes that could indicate material disadvantage within primary care, using a large dataset. Our work is timely as health inequalities have risen since the COVID-19 pandemic, such that other researchers have recently made the case for identifying disadvantaged patients in primary care.^18^ Our attempt to develop such a measure used a wide range of codes. However, without any standardised coding for people’s social and material circumstances in CPRD, it is likely that we missed some codes that indicated disadvantage. Some of the codes included in our binary definition of material disadvantage were clear indicators of disadvantage, such as receipt of means-tested benefits. However, other codes were harder to interpret and may suggest vulnerability or infirmity rather than a lack of material resources (eg, codes that describe neglect of personal hygiene). As poverty is difficult to transition out of, we also assumed a history of material disadvantage when creating our binary definition. That is, if participants had any code for material disadvantage, then they continued to be disadvantaged from that point onwards. However, we recognise that this may not always be the case. Our measure also assumed that participants without one of the specified codes were not experiencing material disadvantage, an approach necessary in order to create a distinction between two populations. The reality is that disadvantage is a gradient rather than binary, and this should be accounted for in future efforts to incorporate a measure of such in primary care.

### Implications and conclusion

This exploratory analysis suggests that there may be merit in having a consensus code to identify persons experiencing material disadvantage in primary care. This is feasible as clinical coding is a dynamic process, with codes recently created to identify unpaid carers for example.^19^ Sparse recording of material disadvantage in primary care represents a missed opportunity to improve population health, given that social circumstances have an important impact on workload, as well as patients’ outcomes and ability to self-manage multiple long-term conditions.^15 20^ Identifying disadvantaged patients might also enable GPs to connect them to appropriate sources of support.^18^ The potential consequences of such a code need to be sensitively considered to ensure that the benefits outweigh any costs at an individual level.

## supplementary material

10.1136/jech-2024-222396 online supplemental file 1

## References

[1] Spiers GF, Liddle JE, Stow D (2022). Measuring older people’s socioeconomic position: a scoping review of studies of self-rated health, health service and social care use. J Epidemiol Community Health.

[2] Lynch JW, Smith GD, Kaplan GA (2000). Income inequality and mortality: importance to health of individual income, psychosocial environment, or material conditions. BMJ.

[3] Alley DE, Soldo BJ, Pagán JA (2009). Material resources and population health: disadvantages in health care, housing, and food among adults over 50 years of age. Am J Public Health.

[4] Barnett K, Mercer SW, Norbury M (2012). Epidemiology of multimorbidity and implications for health care, research, and medical education: a cross-sectional study. The Lancet.

[5] O’Reilly D (2002). Standard indicators of deprivation: do they disadvantage older people?. Age Ageing.

[6] Bradshaw J (2001). Methodologies to measure poverty: more than one is best. york.

[7] Clinical Practice Research Datalink (2022). CPRD aurum may 2022 (version 2022.05.001) data set.

[8] (2023). CPRD Aurum Data Specification Version 3.1.

[9] Robert SA, Cherepanov D, Palta M (2009). Socioeconomic status and age variations in health-related quality of life: results from the national health measurement study. J Gerontol B Psychol Sci Soc Sci.

[10] Office for National Statistics (2020). Population by Index of Multiple Deprivation (IMD) England 2001 to 2019.

[11] Wolf A, Dedman D, Campbell J (2019). Data resource profile: Clinical Practice Research Datalink (CPRD) Aurum. Int J Epidemiol.

[12] Disparities OfHI (2024). Wider determinants of health. https://fingertips.phe.org.uk/profile/wider-determinants.

[13] England N (2024). GP registration digital guide.

[14] Willems SJ, Swinnen W, De Maeseneer JM (2005). The GP’s perception of poverty: a qualitative study. Fam Pract.

[15] McCallum M, MacDonald S (2021). Exploring GP work in areas of high socioeconomic deprivation: a secondary analysis. BJGP Open.

[16] Mercer SW, Watt GCM (2007). The inverse care law: clinical primary care encounters in deprived and affluent areas of Scotland. Ann Fam Med.

[17] Gopfert A, Deeny SR, Fisher R (2021). Primary care consultation length by deprivation and multimorbidity in England: an observational study using electronic patient records. Br J Gen Pract.

[18] Gopal DP, Beardon S, Caraher M (2021). Should we screen for poverty in primary care?. Br J Gen Pract.

[19] NHS England (2022). Coding Unpaid Carers: SNOMED CT.

[20] O’Brien R, Wyke S, Guthrie B (2011). An “endless struggle”: a qualitative study of general practitioners’ and practice nurses’ experiences of managing multimorbidity in socio-economically deprived areas of Scotland. Chron Illn.

