# Supplementary material for: Is it possible to identify populations experiencing material disadvantage in primary care? A feasibility study using the Clinical Practice Research Database
Source: J Epidemiol Community Health. 2024 Sep 3;78(12):e222396. doi: 10.1136/jech-2024-222396 (PMC11671866; doi:10.1136/jech-2024-222396)
Supplement: online supplemental file 1 [file jech-78-12-s001.pdf]

Appendix 1

Medcodeid	Term	SnomedConceptID	Category
12618981000006111	Signposting to benefits advisor	1104561000000107	Benefits
32441000000117	DS1500 form - attendance allowance claim	13571000000107	Benefits
34021000000110	IB113 DLS Incapacity for work form completed	14111000000105	Benefits
34041000000115	IB113 DLS Incapacity for work form sent	14121000000104	Benefits
34061000000119	IB113 DLS Incapacity for work form received	14131000000102	Benefits
34081000000111	IB113 DLS Incapacity for work form paid	14141000000106	Benefits
141281000006119	Sickness benefit	160979005	Benefits
251019010	Sickness benefit	160979005	Benefits
744681000006113	Invalidity pension	160981007	Benefits
251023019	Invalidity pension	160981007	Benefits
251022012	Disablement pension	160981007	Benefits
251024013	Severe disablement allowance	160982000	Benefits
251027018	Attendance allowance	160983005	Benefits
251028011	Receipt of Attendance Allowance	160984004	Benefits
251029015	Mobility allowance	160985003	Benefits
251033010	Industrial disablement benefit	160987006	Benefits
4537401000006117	Industrial injuries disablement benefit	160987006	Benefits
251037011	War disablement pension	160989009	Benefits
251039014	Disability living allowance	160991001	Benefits
4537451000006118	DLA - Disability living allowance	160991001	Benefits
251052016	Widows allowance	160999004	Benefits
251053014	Widowed mother's allowance	161000000	Benefits
251054015	Widows pension	161001001	Benefits
251056018	War widows pension	161003003	Benefits
251058017	Unemployment benefits	161005005	Benefits
251061016	Unemployment benefit NOS	161006006	Benefits
251059013	Unemployment benefit	161006006	Benefits
251069019	Parent's benefits	161014000	Benefits
251072014	One parent benefit	161017007	Benefits
251073016	Child's special allowance	161018002	Benefits
251087016	Low income benefits NOS	161020004	Benefits
251075011	Low income benefits	161020004	Benefits
251078013	Housing benefit	161023002	Benefits
251083017	School meals free	161028006	Benefits
251084011	School fares free	161029003	Benefits
251085012	School uniform free	161030008	Benefits
4537851000006115	Entitled to benefits	161040006	Benefits
12221000000118	IB74 Incapacity benefit	1641000000103	Benefits
12601000000112	DLA 370 Disability living allowance completed	16681000000104	Benefits
265021012	Benefits counselling	171001002	Benefits
1713191000006111	War pensions report	1713191000006107	Benefits
265561015	Attendance allowance medical	171378002	Benefits
265563017	Mobility allowance examination	171379005	Benefits
265562010	Mobility allowance medical	171379005	Benefits
8003721000006115	DS4 attendance allowance examination fee paid	17241000000107	Benefits
1786761000006112	Employment and support allowance	1786761000006108	Benefits
1841411000006116	Employment and Support Allowance form	1841411000006100	Benefits
1841421000006112	ESA113 Employment and Support Allowance form received	1841421000006108	Benefits
1841431000006110	ESA113 Employment and Support Allowance form completed	1841431000006106	Benefits
8006141000006118	Incapacity benefit status	18481000000102	Benefits
4732431000006114	Mobility allowance medical <1.5 hours ex-surgery	184953003	Benefits
4732611000006118	Mobility allowance report - fee paid	185001002	Benefits
285035019	War Pensions report requested	185011009	Benefits
285036018	War Pensions report sent	185012002	Benefits
285037010	Report to benefits agency	185014001	Benefits
8058581000006114	Referral to benefits officer	198771000000100	Benefits
4915861000006113	Benefits education	223433009	Benefits
4915871000006118	Advice about benefits	223433009	Benefits
4924991000006115	Entitlement to benefits uncertain	224195002	Benefits
337179018	Benefits received	224196001	Benefits
337180015	Not receiving all benefits due	224197005	Benefits
4925021000006117	Delay in receiving benefits	224198000	Benefits
4925031000006119	Loss of benefits	224199008	Benefits
4932241000006110	Benefits and pensions	224773006	Benefits
4932251000006112	Social security benefits	224774000	Benefits
4932261000006114	National Health Service benefits	224775004	Benefits
4932271000006119	Local authority benefits	224776003	Benefits
4976581000006115	Application for benefit	228162002	Benefits
4976571000006118	Application for benefit status	228162002	Benefits
4976601000006113	Receipt of benefit	228163007	Benefits
4988851000006115	Means tested benefit	229061000	Benefits
4988861000006118	Income support	229062007	Benefits
25931000000111	MY22 - mobility allowance form	23461000000107	Benefits
8011161000006114	MY22 - mobility allowance form	23461000000107	Benefits
251042015	Sickness/invalid benefit NOS	266975006	Benefits
251041010	Benefits - other, specified	266975006	Benefits

Appendix 1

397805011	Sickness/invalidity benefit	266975006	Benefits
1694071000006115	DS1500 attend allowance NOS	270377001	Benefits
405024012	DS4 attend allowance exam NOS	270377001	Benefits
631671000006118	DS4-attendance allowance exam	270377001	Benefits
5516271000006114	DS4 attendance allowance examination	270377001	Benefits
405027017	Mobility allowance claim rep.	270389004	Benefits
251057010	Widows benefits NOS	270470003	Benefits
53421000006114	Widows pensions	270470003	Benefits
53381000006116	Widows allowances	270470003	Benefits
405075010	Widows benefits	270470003	Benefits
5517731000006118	DS1500 attendance allowance exam	270841001	Benefits
418915011	Invalidity benefit	281019004	Benefits
700511000006118	Disability living allowance medical examination fee paid	307521000000103	Benefits
700521000006114	Disability living allowance medical examination fee unpaid	307531000000101	Benefits
8099531000006114	Disability living allowance medical examination form sent off	307561000000106	Benefits
451944019	War pensions report	308619006	Benefits
5983321000006117	Benefits, entitlements and rights	308911007	Benefits
284868018	Social security report status	309029009	Benefits
5984051000006110	War pensions report status	309031000	Benefits
453975018	Benefits agency reports fit for work	310402006	Benefits
454029019	Report requested by benefits agency	310456004	Benefits
8103891000006118	Social services financial assessment	323781000000106	Benefits
735851000000113	Benefits agency reports unfit for work	374171000000109	Benefits
851921000006119	Referral to benefits advisor	514521000000105	Benefits
7982421000006119	DS1500 Attendance allowance examination payment claim	5391000000104	Benefits
13201000000118	Mobility allowance claim report status	5741000000108	Benefits
25941000000119	Mobility allowance exam admin	715231000000105	Benefits
25601000000114	Attendance allowance examination administration	715271000000107	Benefits
8230041000006115	Allowance	749011000000104	Benefits
1714811000000118	DS1500 Disability living allowance report declined	768381000000107	Benefits
8248711000006117	Receiving income-related employment and support allowance	789851000000101	Benefits
1775291000000119	Receiving carer allowance	794111000000107	Benefits
13161000000114	Mobility allowance report completed	8121000000105	Benefits
7988731000006113	Mobility allowance report sent off	8131000000107	Benefits
13221000000110	Attendance allowance form completed	8151000000100	Benefits
2128041000000115	Employment and support allowance	816691000000101	Benefits
1863651000006113	Referral to Benefits Agency	818881000000103	Benefits
2170171000000114	Employment and support allowance status	835611000000109	Benefits
851491000006111	Benefits assessed	851491000006107	Benefits
851841000006116	Benefits advice	851841000006100	Benefits
852181000006113	Receiving all benefits	852181000006109	Benefits
909111000006112	[RFC] Social welfare	909111000006108	Benefits
2339381000000110	Personal Independence Payment claim form	910011000000105	Benefits
2339471000000119	Personal Independence Payment claim form completed	910051000000109	Benefits
2347831000000111	Personal Independence Payment applied for	913641000000108	Benefits
2347871000000113	Personal Independence Payment	913661000000109	Benefits
923491000006118	Benefits counselling	923491000006102	Benefits
1921301000006115	Benefits agency reports unfit for work but fit note no longer needed	925481000000108	Benefits
159541000006112	Sacked from job	105496009	Employment
217551000000117	Sacked from work	105496009	Employment
4119741000006119	Dismissed from job	105496009	Employment
8437331000006113	Care leaver	1064671000000107	Employment
12116491000006117	Referral to employment support service	1098041000000101	Employment
12116501000006113	Employment support	1098051000000103	Employment
9781000000118	Dismissed - notice given	1221000000107	Employment
14282141000006111	Employment status : Unemployed and actively seeking work	14282141000006107	Employment
14282161000006110	Employment status : Long-term sick/disabled/receiving sickness and disabili	14282161000006106	Employment
14282181000006117	Employment status : Not receiving sickness/disability benefits and not work	14282181000006101	Employment
250175015	Social group 5 - unskilled	160486005	Employment
250794019	Partner works after retirement	160856005	Employment
250817016	Partner unemployed	160875000	Employment
250858011	Recently unemployed	160899000	Employment
250859015	Chronic unemployment	160900005	Employment
250861012	Redundant	160901009	Employment
4536631000006114	Made redundant	160901009	Employment
250862017	Works after retirement	160902002	Employment
251060015	Redundancy payment	161007002	Employment
9681000000114	No employment contract	16571000000102	Employment
9721000000119	Employ. contract out of date	16581000000100	Employment
9791000000116	Dismissed - immediate	16601000000109	Employment
1746171000006114	GPPAQ not in employment	1746171000006105	Employment
1876841000006111	Economic activity status - perm. sick/disabled	1876841000006107	Employment
1979561000006112	Socio-economic classification code (stop smoking): Never worked or unemp	1979561000006108	Employment
1979581000006119	Socio-economic classification code (stop smoking): Home carer	1979581000006103	Employment
1979591000006116	Socio-economic classification code (stop smoking): Sick or disabled and una	1979591000006100	Employment
1979951000006115	Socio-economic classification code (stop smoking): Routine and manual occ	1979951000006104	Employment
4927051000006119	In paid casual work	224364001	Employment

4927061000006117	In paid seasonal work	224365000	Employment
4927151000006115	On a pathway to employment scheme	224371006	Employment
4927211000006110	Works irregularly	224376001	Employment
4927261000006113	Job seeking history	224380006	Employment
4927271000006118	Seeking work	224381005	Employment
4927311000006118	Registered with job center	224384002	Employment
4927301000006116	Registered with job centre	224384002	Employment
4927341000006119	Registered with disability employment advisor	224387009	Employment
4928391000006112	Unfair dismissal from job	224474004	Employment
5182801000006113	Blind and partially sighted rehabilitation - employment placement	243113006	Employment
397781013	Loss of job	266956001	Employment
5496391000006114	Lost job	266956001	Employment
397786015	Poor work record	266960003	Employment
460747011	[V]Threat of job loss	276074009	Employment
412067018	Excess sick leave	276076006	Employment
5653241000006110	Parent unemployed	281577004	Employment
5654251000006114	Unemployed father	281662004	Employment
5654261000006111	Unemployed mother	281663009	Employment
5897411000006118	Ability to undertake job application activities	301718001	Employment
5897521000006113	Difficulty undertaking job application activities	301723001	Employment
6025441000006119	Redundancy counselling	313084001	Employment
250927014	Finding of job details	365526009	Employment
6271821000006117	Job details - finding	365526009	Employment
460996012	[V]Unemployment	73438004	Employment
502435010	Unemployed	73438004	Employment
3694101000006118	Without employment	73438004	Employment
3694131000006114	U/E - Unemployed	73438004	Employment
3694111000006115	Out of work	73438004	Employment
460745015	[V]Problems related to employment and unemployment	75148009	Employment
502935015	Employment problem	75148009	Employment
13490511000006117	Care leaver	770347003	Employment
8247381000006112	Referral for employment support	787421000000102	Employment
2170011000000117	Referral to employability service	835531000000102	Employment
8285121000006112	Employment support indicated	844391000000109	Employment
141981000000118	Epilepsy restricts employment	93141000000105	Employment
141991000000116	Epilepsy prevents employment	93151000000108	Employment
2441101000000110	NEET - Not in employment, education or training	957481000000103	Employment
2441091000000119	Not in employment, education or training	957481000000103	Employment
8367221000006118	Current View Education,Employment,Training score - attainment difficulties	987231000000105	Employment
8367231000006115	Current View Education,Employment,Training score - attendance difficulties	987241000000101	Employment
1876851000006113	Economic activity status - temp. sick/disabled	1876851000006109	Emtloyment
336165015	Environmental pollution	102411008	Environment
460765010	Exposure to polluted air	102424008	Environment
460756015	Exposure to polluted air, occupational	102425009	Environment
284471016	Reg. in deprived area NOS	184165003	Environment
284467019	Registered in deprived area	184165003	Environment
179461000006113	Registered in deprived area - high	184166002	Environment
4924341000006113	Local environment and neighbourhood details	224153006	Environment
460774012	[V]Problem related to physical environment, unspecified	243796009	Environment
460771016	[V]Other problems related to physical environment	243796009	Environment
460763015	[V]Problems related to physical environment	243796009	Environment
959051000006118	Inadequate/unavailable resources	959051000006102	Environment
959411000006110	High pollution level	959411000006106	Environment
959461000006113	Inadequate/unsafe play/exercise areas	959461000006109	Environment
980971000006113	Inadequate space/resources to foster health	980971000006109	Environment
169620017	Homeless family	105526001	Housing
460968018	[V]Inadequate housing	105531004	Housing
460776014	[V]Problems related to housing and economic circumstances	105531004	Housing
371003018	Housing very unsatisfactory	105531004	Housing
397753019	Inadequate housing NOS	105531004	Housing
291191016	Housing unsatisfactory	105531004	Housing
504820019	Housing problem	105531004	Housing
452431000006113	Accommodation unsuitable	105531004	Housing
217521000000110	Housing unsatifactory	105531004	Housing
398074012	Inadequate housing	105531004	Housing
291210013	Overcrowded in house	105532006	Housing
1227736010	[V]Lack of heating	105535008	Housing
291291013	Lack of heat in house	105535008	Housing
8452221000006119	Referred by homeless drop-in centre	1077211000000104	Housing
2714671000000111	Signposting to housing support service	1083301000000108	Housing
2733781000000113	Referral to homeless advocacy service	1091381000000101	Housing
2741511000000113	Declined to disclose housing status	1094411000000102	Housing
2743651000000110	Living temporarily with friends	1095421000000107	Housing
2743791000000114	Provision of community outreach care for homeless	1095491000000105	Housing
12619001000006114	Signposting to homeless support service	1104581000000103	Housing
250503019	Homeless single person	160700001	Housing
250518018	Lack of space in house	160706007	Housing

Appendix 1

250541014	Inappropriate housing	160721001	Housing
4535011000006117	Living conditions unsuited to needs	160721001	Housing
4535001000006115	Housing unsuited to needs	160721001	Housing
828681000006119	Housing dependency scale	160723003	Housing
174891000006119	Lives in sheltered housing	160729004	Housing
250562010	Lives in a welfare home	160736003	Housing
250574014	Living in B&B accommodation	160744003	Housing
4535201000006114	Lives in bed and breakfast accommodation	160744003	Housing
250578012	Living in lodgings	160745002	Housing
250580018	Lives in a bedsit	160746001	Housing
250579016	Living in bedsitter	160746001	Housing
250587015	Housing problems - eviction	160751007	Housing
250589017	Eviction notice served	160752000	Housing
250590014	Evicted forcibly from house	160753005	Housing
250591013	Tenant evicted	160754004	Housing
828551000006114	House rented from housing association	160939001	Housing
251110012	At risk hypothermia	161052004	Housing
412961000006117	[X]Other problems related to housing & econom circumstances	161152002	Housing
226421000000116	Homeless enhanced services administration	166301000000107	Housing
226561000000111	Homeless enhanced service completed	166461000000103	Housing
10321000000112	Rent and rates payment status	18541000000109	Housing
1856511000006113	Damp aggravates symptom	1856511000006109	Housing
285904010	On urgent housing list	185955000	Housing
285905011	On housing list	185956004	Housing
285906012	Awaiting housing improvement	185957008	Housing
285907015	Housing problem solved	185960001	Housing
1859751000006114	Lives in mobile accommodation	1859751000006105	Housing
1859831000006110	Lives in caravan	1859831000006106	Housing
1859901000006113	Lives in sublet accommodation	1859901000006109	Housing
1859911000006111	Housing ownership and tenure: not known	1859911000006107	Housing
1872181000006110	Accom status - tenant - local authority/arms length management	1872181000006106	Housing
1872191000006113	Accom status - tenant - housing association	1872191000006109	Housing
1872201000006111	Accom status - tenant - private landlord	1872201000006107	Housing
1872221000006118	Accom status - homeless	1872221000006102	Housing
1872231000006115	Accom status - rough sleeper	1872231000006104	Housing
1872241000006113	Accom status - squatting	1872241000006109	Housing
1872251000006110	Accom status - night shelter/emergency or direct access hostel	1872251000006106	Housing
1872261000006112	Accom status - sofa surfing	1872261000006108	Housing
1872271000006117	Accom status - temporary accommodation by local authority	1872271000006101	Housing
1872281000006119	Accom status - staying with friends/family as a short term guest	1872281000006103	Housing
1872291000006116	Accom status - other homeless	1872291000006100	Housing
1872451000006111	Accom status - Foyer - ages 16-25 homeless or in housing need	1872451000006107	Housing
1872601000006114	Accom status - mobile accommodation	1872601000006105	Housing
1892151000006112	CPU risk factor (parent/carer) - housing/accommodation	1892151000006108	Housing
1894451000006110	No longer homeless	1894451000006106	Housing
1895861000006114	Child affected by significant housing need	1895861000006105	Housing
1934521000006115	Parenting intervention delivered by housing association	1934521000006104	Housing
1950961000006110	Referral to homeless team	1950961000006106	Housing
1951001000006118	Patient discharge, sofa surfing	1951001000006102	Housing
1951041000006116	Discharge to rented accommodation	1951041000006100	Housing
1951051000006119	Discharge to council accommodation	1951051000006103	Housing
1951071000006112	Discharge to bed and breakfast accommodation	1951071000006108	Housing
1951091000006113	Referral to rough sleeper outreach team	1951091000006109	Housing
1951101000006119	Referral to day centre for homeless	1951101000006103	Housing
1951111000006116	Referral to support worker for homeless	1951111000006100	Housing
1996631000006115	Support to obtain accommodation or housing	1996631000006104	Housing
1999451000006118	Prevention of accommodation loss	1999451000006102	Housing
2002641000006117	Referral to homeless advocacy service	2002641000006101	Housing
2002921000006119	Assertive outreach in the care of the homeless	2002921000006103	Housing
2004001000006118	Declined referral to homeless advocacy service	2004001000006102	Housing
636251000000114	Asthma trigger - damp	201201000000105	Housing
8061031000006119	Asthma trigger - damp	201201000000105	Housing
2015681000006119	Problem with accommodation	2015681000006103	Housing
337197014	Lives in rented accommodation	224213000	Housing
4925211000006118	Lives in furnished rented accommodation	224214006	Housing
4925221000006114	Lives in unfurnished rented accommodation	224215007	Housing
4925361000006113	No fixed abode	224226001	Housing
4925371000006118	NFA - No fixed abode	224226001	Housing
337216012	Living rough	224228000	Housing
4925421000006113	Temporary shelter arrangements	224230003	Housing
4925501000006119	Status of waiting list for accommodation	224238005	Housing
4925521000006112	On waiting list for accommodation	224239002	Housing
4925631000006114	Lack of space for privacy in accommodation	224248007	Housing
4925681000006110	Rising damp in home	224252007	Housing
4925711000006111	Mould growth in home	224255009	Housing
4925731000006117	Mould on surfaces in home	224256005	Housing
4925841000006115	Adequacy of living space	224263005	Housing

4925941000006113	Dirty home	224270005	Housing
4930951000006115	Fixed-site mobile home	224665006	Housing
4931071000006116	Hostel for the homeless	224675009	Housing
4932341000006115	Dirty environment	224783005	Housing
4932351000006118	Damp environment	224784004	Housing
4939561000006119	Housing assessment	225340009	Housing
343641000006117	[V]Other problems related/housing and economic circumstances	243796009	Housing
405061000000116	Living in temporary housing	247521000000104	Housing
5385491000006118	Hostel	257628001	Housing
5385641000006112	Mobile home	257641002	Housing
250509015	Housing lack NOS	266935003	Housing
397751017	Housing lack	266935003	Housing
397752012	House in poor repair	266936002	Housing
397756010	Living in hostel	266939009	Housing
5496121000006110	Lives in residential hostel	266939009	Housing
334561015	[X]Lack of water, occurrence at home	269703005	Housing
334560019	[X]Lack of water	269703005	Housing
285909017	Awaiting housing NOS	271364002	Housing
500881000006110	Awaiting housing or re-housing	271364002	Housing
5523881000006110	Finding relating to awaiting housing or re-housing	271364002	Housing
411615019	Housing - awaiting action	275643002	Housing
5687821000006116	Residential caravan park	284451009	Housing
5697331000006111	Cold environment	285123000	Housing
341191000006116	[V]Housing, household and economic circumstances	288531000119103	Housing
5903521000006114	Housing, local environment and transport detail	302149003	Housing
453150016	Unsatisfactory living conditions	308899009	Housing
453151017	Slum housing	309683008	Housing
6003681000006117	Housing report status	310853003	Housing
454372016	Housing report	310854009	Housing
454373014	Housing report requested	310855005	Housing
454374015	Housing report sent	310856006	Housing
454375019	Housing report paid	310857002	Housing
6012761000006113	Incapacity benefit	312094004	Housing
7975861000006110	DS4 attendance allowance examination payment claim less than 1.5 hours,	3181000000103	Housing
7975951000006116	DS4 attendance allowance examination fee unpaid	3201000000104	Housing
485356010	Homeless	32911000	Housing
6260921000006111	Feature of homelessness	364701009	Housing
6260931000006114	Details of awaiting housing or re-housing	364702002	Housing
488727013	Characteristics of home environment - finding	365512000	Housing
752321000000118	Sofa surfer - person of no fixed abode	381751000000106	Housing
1480173012	Housing related procedures	385985008	Housing
91151000000111	Referral to housing department	38921000000104	Housing
1786092012	Damp housing	398081001	Housing
1777647018	Damp in house	398081001	Housing
6597501000006119	Damp in home	398081001	Housing
460967011	[V]Lack of housing	398341000000102	Housing
12458771000006118	[V]Lack of housing	398341000000102	Housing
6723681000006110	Living temporarily with relatives	406132007	Housing
2576303013	Housing education, guidance, and counselling	410297008	Housing
6795881000006115	Housing case management	410341008	Housing
7025931000006117	Dirty living conditions	424415008	Housing
460770015	Water supply insufficient	424466003	Housing
757521000000112	Length of time homeless	442244004	Housing
8186591000006114	Lives in housing association rented accommodation	491761000000102	Housing
2286501000000119	Under care of homeless advocacy service	702526004	Housing
1716301000000118	Housing assessment completed	704045001	Housing
8249091000006117	Referral by housing service	790331000000107	Housing
285903016	Letter written to housing	807261000000105	Housing
12618181000006113	At risk of homelessness	82531000000100	Housing
3848311000006110	Living in mobile home	82876001	Housing
14338741000006113	Lives in mobile home	82876001	Housing
3866031000006114	Hypothermia caused by cold environment	83966006	Housing
3866021000006111	Hypothermia due to cold environment	83966006	Housing
856001000006119	Housing conditions	856001000006103	Housing
2312161000000117	Discharge from homeless advocacy service	898811000000106	Housing
905961000006114	[RFC] Housing and welfare advice	905961000006105	Housing
909331000006116	[RFC] Housing assessment	909331000006100	Housing
2341611000000113	No hot water in home	910981000000102	Housing
939591000006119	Housing status	939591000006103	Housing
940741000006110	Homeless within the past year	940741000006106	Housing
958501000006115	Inadequate food storage/disposal	958501000006104	Housing
958531000006111	Inadequate water supply	958531000006107	Housing
958781000006117	Inadequate sewage disposal	958781000006101	Housing
958811000006115	Inadequate laundry facilities	958811000006104	Housing
958871000006112	Inadequate heating/cooling	958871000006108	Housing
959211000006114	Inadequate/crowded living space	959211000006105	Housing
959371000006114	Homeless	959371000006105	Housing

968381000006110	Has no fixed abode	968381000006106	Housing
982341000006115	At risk of hypothermia	982341000006104	Housing
982361000006116	Damp/condensation	982361000006100	Housing
982961000006117	Lack of personal care facilities	982961000006101	Housing
994091000006118	Living in temporary housing	994091000006102	Housing
8428631000006118	Economic wellbeing intervention	1054421000000104	Income
169689019	Bankruptcy	105577008	Income
1227737018	[V]Poverty NOS	11403006	Income
460780016	[V]Extreme poverty	11403006	Income
19738011	Poverty	11403006	Income
2681741000006112	Financially poor	11403006	Income
1227738011	[V]Economic problem	160932005	Income
460969014	[V]Inadequate material resources	160932005	Income
250937016	Financial problem	160932005	Income
250938014	In debt	160933000	Income
250939018	Financial circumstances change	160934006	Income
283508015	Economic rehab. measures	183439000	Income
816951000006114	Has free prescriptions - low income	184782008	Income
1892191000006118	CPU risk factor (parent/carer) - poverty/financial	1892191000006102	Income
1898181000006118	Born into single parent family	1898181000006102	Income
1911671000006114	Barrier to parental engagement - finance	1911671000006105	Income
1979211000006112	Referred for social/financial support	1979211000006108	Income
2008441000006116	Child affected by poverty	2008441000006100	Income
36839019	Single parent family	21959005	Income
4924541000006116	Financial circumstances	224164009	Income
4924621000006110	No household income	224169004	Income
4924631000006113	Low household income	224170003	Income
4924691000006112	No personal income	224174007	Income
4924701000006112	Low personal income	224175008	Income
4924871000006110	Sufficiency of income for needs	224189003	Income
4924911000006113	Income insufficient to meet needs	224191006	Income
223711000000111	Shop lifting	248035003	Income
370296019	Shoplifting	248035003	Income
397763010	Single parent	266943008	Income
142681000006113	Single parent family - mother	266977003	Income
412068011	Business ceased - financial	276077002	Income
1591621000006118	Destitute	284477001	Income
5883391000006112	Unable to manage personal financial activities	300683002	Income
5883431000006118	Difficulty managing personal financial activities	300686005	Income
5883501000006110	Difficulty with money management	300692004	Income
1780263012	Needs help managing own financial affairs	401058009	Income
6729071000006118	Income needs assessment	406536004	Income
2549598015	At risk of financial/material mismanagement	417051004	Income
460781017	Low income	424860001	Income
460782012	[V]Insufficient social insurance and welfare support	456281000000100	Income
12458681000006114	[V]Insufficient social insurance and welfare support	456281000000100	Income
7680551000006114	Referral to financial service	710913009	Income
1756731000000119	Wishes to see money advisor	785511000000101	Income
958451000006117	Low/no income	958451000006101	Income
958461000006115	Difficulty with money management	958461000006104	Income
958471000006110	Able to buy only necessities	958471000006106	Income
958481000006113	Difficulty buying necessities	958481000006109	Income
2508981000000114	Personal financial circumstances reviewed	986691000000106	Income
256145015	Neglected appearance	102892007	Neglect
252576015	Hunger pain	162043005	Neglect
253445019	Irregular meal frequency	162552006	Neglect
253447010	Rarely has breakfast	162554007	Neglect
324687017	Effects of hunger NOS	212966005	Neglect
324683018	Effects of hunger	212966005	Neglect
324690011	Deprivation of water	212971003	Neglect
901301000006111	Accident neglect:hunger/thirst	217632008	Neglect
452251000006119	Accidents due to hunger, thirst, exposure and neglect	217632008	Neglect
334549011	[X]Lack of food, occurrence at home	217640002	Neglect
334548015	[X]Lack of food	217640002	Neglect
329877015	Accident due to hunger	217640002	Neglect
329875011	Accident due to lack of food	217640002	Neglect
5252341000006111	Neglect of clothes	248055002	Neglect
5252351000006113	Neglect of personal hygiene	248056001	Neglect
5252361000006110	Neglect of physical health	248057005	Neglect
5252371000006115	Neglect of dental care	248058000	Neglect
5253681000006117	Inadequately dressed	248161002	Neglect
5281431000006114	Fastening of footwear inadequate	250160007	Neglect
5281501000006117	Material used to construct footwear inadequate	250165002	Neglect
404240011	Accident due to thirst	269703005	Neglect
404239014	Accident due to lack of water	269703005	Neglect
324684012	Deprivation of food	287519002	Neglect
494331000000112	Patient not registered with a dentist	413314002	Neglect

Appendix 1

6839591000006112	Patient not registered with dentist	413314002	Neglect
1692561000000118	Rarely has evening meal	449279001	Neglect
899851000006114	Deprivation of food	696931000000104	Neglect
1692101000000112	Rarely has lunch	763191000000104	Neglect
1748121000000117	Replaces meals with drinks	781911000000108	Neglect
419821000006111	[X]Persons with potential health hazards related to socioeconomic and psyc	161152002	Other socioeconomic measures
251263011	Poor social circumstances	161154001	Other socioeconomic measures
283509011	Other socioeconomic measures	183441004	Other socioeconomic measures
12716881000006110	Other socioeconomic measures	183441004	Other socioeconomic measures
2005971000006110	Exposure to determinant of health inequality: Social	2005971000006106	Other socioeconomic measures
325630015	[X]Effect of deprivation, unspecified	285153007	Other socioeconomic measures
636771000006116	Effect of deprivation, unspecified	285153007	Other socioeconomic measures
460784013	Problem related to social environment	288531000119103	Other socioeconomic measures
5903501000006116	Legal, financial, employment and socioeconomic history detail	302148006	Other socioeconomic measures
325625019	[X]Other effects of deprivation	417163006	Other socioeconomic measures
2652211000000114	Signposting to Citizens Advice	1053761000000102	Support service
8439191000006118	Referral to food bank	1066291000000102	Support service
8439181000006116	Referral to foodbank	1066291000000102	Support service
2714901000000112	Signposting to food bank	1083401000000103	Support service
2719371000000116	Signposting to support service for carers	1085401000000104	Support service
1938021000006119	Food bank voucher issued to patient	1938021000006103	Support service
405361000000118	Refer to Citizens Advice Bureau	247671000000104	Support service
752841000000117	Referral to affordable warmth programme	382011000000100	Support service
1961531000006113	Referral to community meals service	713109004	Support service
851911000006110	Referral to citizens advice bureau	851911000006106	Support service
1868341000006113	Provision of written information about Affordable Warmth programme	860251000000109	Support service
250495019	Transport economic problems	160695008	Transport
397197011	Invalid transport facilities	266738008	Transport
450289016	No car	307109002	Transport
8220881000006115	Fuel poverty	719781000000101	Transport
